# Efficacy of transcatheter patent foramen ovale closure for drug-resistant migraine: initial experience in Japan and long-term outcome

**DOI:** 10.1007/s12928-025-01135-4

**Published:** 2025-06-04

**Authors:** Teiji Akagi, Yoichi Takaya, Takashi Miki, Rie Nakayama, Koji Nakagawa, Mitsuki Nakashima, Yoshiaki Takahashi, Nozomi Hishikawa, Shinsuke Yuasa

**Affiliations:** 1 Department of Cardiovascular Medicine, Graduate School of Medicine, Dentistry and Pharmaceutical Sciences, Okayama University, 2-5-1 Shikata-cho, Kita-ku, Okayama, 700-8558 Japan; 2 Department of Neurology, Graduate School of Medicine, Dentistry and Pharmaceutical Sciences, Okayama University, 2-5-1 Shikata-cho, Kita-ku, Okayama, 700-8558 Japan; 3 Present Address: The Sakakibara Heart Institute of Okayama, 2-5-1 Nakai-cho, Kita-ku, Okayama, 700-0804 Japan

**Keywords:** Patent foramen ovale, Migraine, Headache, Stroke, Catheter

## Abstract

This study evaluates the efficacy and safety of transcatheter patent foramen ovale (PFO) closure for the treatment of drug-resistant migraine in Japan. Previous studies have suggested a potential benefit for migraine with aura, although large-scale trials in the United States and Europe have failed to confirm efficacy as a primary endpoint. The study included 27 patients (mean age 36.4 years, 15 female, 21 with aura) who had more than two migraine attacks per month despite medication. All had PFO confirmed by transesophageal echocardiography and underwent transcatheter closure with the Amplatzer PFO Occluder. Patients were followed up to 12 months with migraine severity monitored by headache specialist. The procedure was successful and without complications in all cases. One patient required a larger occluder (35 mm) due to the size of PFO. At 12 months, 22 of 27 (81%) patients reported either complete resolution or improvement of migraine. Specifically, 10 of 21 (48%) patients with aura experienced complete resolution of migraine at one year. Patients without aura had a lower response rate, with only one case of complete resolution. Despite limitations such as the lack of a control group and potential patient selection bias, the study demonstrated that PFO closure may provide significant relief for patients with drug-resistant migraine, particularly those with aura. These findings support further investigation to better define its clinical indications and potential benefits.

**Graphical abstract:**

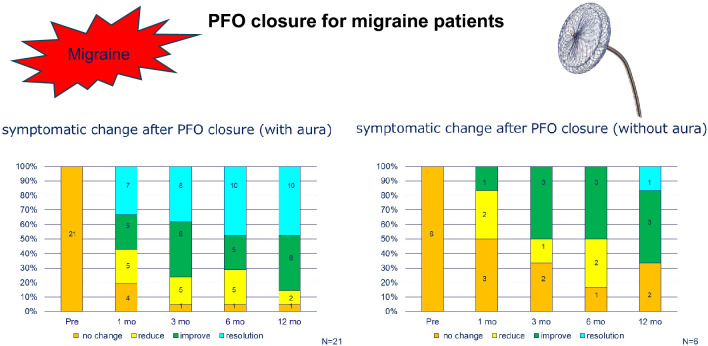

## Introduction

In recent years, patent foramen ovale (PFO) has received increasing attention in the field of structural interventions. Three randomized controlled trials published in 2017 demonstrated that transcatheter PFO closure significantly reduced the recurrence of ischemic stroke in patients under 60 years old compared with conventional medical therapy [1–3]. In Japan, transcatheter closure of PFO was approved as an insurance-covered treatment in December 2019 for secondary prevention of cryptogenic stroke, and clinical experience has been accumulating since then [4].

It has long been reported that patients with cryptogenic cerebral infarction due to PFO often suffer from migraine, and some patients empirically experience dramatic improvement in their migraine symptoms after catheter closure of PFO [5]. Consequently, three randomized controlled trials of transcatheter PFO closure for migraine treatment were conducted alongside clinical trials for recurrent cerebral infarction prevention [6–8]. However, none of these studies could not confirm the efficacy as a primary endpoint. As a result, transcatheter PFO closure is not currently recommended by guidelines in Europe or the United States and is only used on a limited compassionate use basis [9]. In Japan, the Japanese Headache Society has issued a statement on transcatheter PFO closure for migraine, noting that while there is a suspected association between migraine with aura and PFO, the effectiveness of the procedure has not been confirmed, it is not currently recommended, and it is not covered by health insurance [10].

Migraine affects 8–13% of adults aged 15 years and older and is estimated to cause an economic loss of approximately 300 billion yen annually in Japan [11, 12]. Recently, monoclonal antibodies (mAbs) targeting calcitonin gene-related peptide (CGRP) or its receptor have revolutionized migraine treatment. These medications are designed to prevent migraine attacks by inhibiting the CGRP pathway, which plays a key role in migraine pathophysiology. Overall, CGRP-targeting mAbs represent a significant advancement in migraine management, particularly for patients with frequent or refractory migraines. However, cost, administration route, and long-term effects remain important considerations in their use [12–14]. Despite such emergence of innovative treatments, a fundamental solution remains elusive. Among Japanese patients who have suffered ischemic stroke, some with a history of migraine have reported marked improvement or resolution of symptoms following transcatheter PFO closures aimed at preventing recurrent stroke or atrial septal defect closure [15]. Thus, the possibility remains that transcatheter PFO closure may be effective for the treatment of migraine. Clinical experience with transcatheter PFO closure for migraine treatment in Japan is extremely limited. This paper investigates the efficacy, safety, and long-term effects of transcatheter PFO closure in migraine patients refractory to medical treatment.

## Methods

### Patients

Subjects included patients who sought transcatheter PFO closure due to inadequate therapeutic benefit from conventional medical treatments. Patients were interviewed by a neurologist specializing in headache treatment (YT and NH) to assess headache type, migraine frequency, and the presence or absence of aura according to the criteria of International Classification of Headache Disorders, 3rd edition [16]. The presence and morphology of PFO were evaluated using transthoracic and transesophageal echocardiography [17–19]. The same neurologist conducted follow-up interviews throughout the study to evaluate migraine symptoms. The inclusion criteria in this study were:

(1) Age: 16–69 years, (2) migraine occurrence more than twice per month, (3) PFO detected by contrast bubble study on transesophageal echocardiography, (4) resistance to conventional migraine medication, including triptans, (5) no history of ischemic stroke confirmed by MRI, and (6) patient preference for transcatheter closure.

### Transesophageal echocardiography

Assessment of PFO followed previously reported methods [18, 20]. However, this study was conducted before the concept of high-risk PFO was established, limiting detailed morphologic evaluations.

### Transcatheter PFO closure

All procedures were performed under general anesthesia with transesophageal echocardiographic monitoring. Aspirin (100 mg) was administered 24 h before the procedure, and clopidogrel (50 mg) was added afterward for 1 month [21]. Aspirin was discontinued 6 months post-procedure. Follow-up visits were scheduled at 1, 3, 6, and 12 months. Patients were free to choose or discontinue migraine prophylaxis.

### Neurological evaluation and treatment efficacy

The following categories were defined based on interviews and headache diaries:

[No change]: no change in headache frequency or severity.

[Reduce]: reduced headache frequency with continued migraine attacks requiring medication.

[Improve]: rare migraine attacks not requiring medication.

[Resolution]: complete resolution of migraines and discontinuation of medication.

At the time of this study, the Amplatzer PFO Occluder was not approved in Japan and was privately imported. All medical expenses were paid by the patients. The study protocol was approved by the Ethics Committee of Okayama University (UMIN000017216) and complied with the Declaration of Helsinki and ICH-GCP guidelines.

## Subjects

**Fig. 1:**
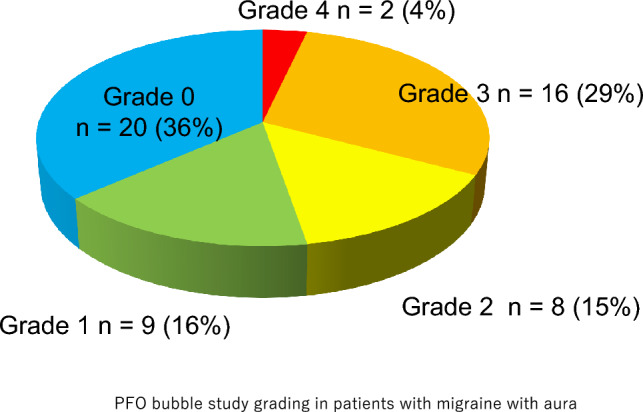
PFO bubble study grading in patients with migraine with aura

Out of 152 headache patients, 50 (33%) tested positive for the bubble study. Among 55 patients with migraine with aura, 35 (64%) tested positive (Fig. 1). After being informed of treatment options, 28 patients underwent transcatheter PFO closure. One patient was later diagnosed with cluster headache and excluded from analysis (Table 1).

**Table 1:** Patient’s profile at enrollment (*n* = 28)

Age at procedure: median (range)	36.4 years (18–63)
Sex:	Female: 15, male: 13
Age at onset of migraine: median (range)	14.4 years (6–30)
Frequency of migraine: median times(range)	8.0/month (0.3–30)
Type:	Migraine with aura (21) Migraine without aura (6) Other type (cluster headache: 1)
Polypharmacy (more than 3 types of headache medications)	All (100%)

Among the 27 remaining patients, 21 had migraine with aura, and 6 had migraine without aura. The cohort included 15 females and 12 males, with a mean age of 36.4 years (range: 17–63). All patients had histories of multiple migraine medications. Twelve patients had atrial septal aneurysms, and 11 had high-risk PFO [15] (PFO score $\ge 2)$(Table 2).

**Table 2:** Patient’s profile (case #1–21: migraine with aura, #22–27: migraine without aura)

#	Age	Sex	Frequency (month)	Aura	TTE shunt grade	PFO height	ASA	PFO risk score	1 month	3 months	6 months	1 year
1	18	M	10	+	1	4	0	2	Improve	Resolution	Resolution	Resolution
2	63	F	8	+	2	1	0	0	Improve	Resolution	Resolution	Improve
3	38	M	5	+	4	1	0	1	Improve	Improve	Improve	Improve
4	32	F	0.3	+	3	1	1	2	Reduce	Reduce	Reduce	Resolution
5	21	F	0.5	+	2	NA	0	0	Resolution	Resolution	Resolution	Improve
6	51	F	30	+	4	1	0	2	Reduce	Improve	Improve	Resolution
7	53	F	2	+	3	1	1	2	Resolution	Improve	Reduce	Reduce
8	50	F	10	+	4	1	1	4	Reduce	Reduce	Reduce	Improve
9	36	F	3	+	3	2	0	1	Resolution	Improve	Resolution	Improve
10	17	F	1	+	3	1	0	1	Improve	Reduce	Resolution	Resolution
11	33	M	10	+	3	2	1	3	No change	Improve	Improve	Improve
12	17	F	30	+	3	1	1	1	No change	Reduce	Reduce	Improve
13	45	F	5	+	2	2	1	1	Reduce	Improve	Improve	Resolution
14	23	M	8	+	2	2	1	1	Improve	Resolution	Resolution	Resolution
15	22	M	2	+	2	1	0	1	Resolution	Resolution	Resolution	Resolution
16	45	M	2	+	3	3	1	2	Resolution	Resolution	Resolution	Resolution
17	18	M	8	+	2	1	0	0	No change	Improve	Reduce	Reduce
18	42	M	2	+	2	2	1	1	Resolution	Resolution	Resolution	Resolution
19	22	M	8	+	3	NA	0	1	Resolution	Resolution	Resolution	Resolution
20	37	F	12	+	2	2	1	3	No change	No change	No change	No change
21	33	F	16	+	2	3	1	3	Reduce	Improve	Improve	Improve
22	32	M	6	−	3	2	0	1	Reduce	Improve	Improve	Improve
23	55	F	15	−	3	5	0	2	No change	Improve	Improve	Resolution
24	36	M	12	−	4	2	0	1	No change	No change	Reduce	Improve
25	40	F	7	−	2	3	1	2	Improve	Improve	Improve	Improve
26	56	M	9	−	3	2	0	1	Reduce	Reduce	Reduce	No change
27	31	F	16	−	3	1	0	1	No change	No change	No change	No change

## Results

All procedures were successfully completed without complications. In all cases except case 20, a 25-mm Amplatzer PFO Occluder was implanted, and complete PFO closure was confirmed. In case 20, a 35-mm Amplatzer PFO device was used due to a large PFO, but a residual shunt was observed after 12 months.

**Fig. 2:**
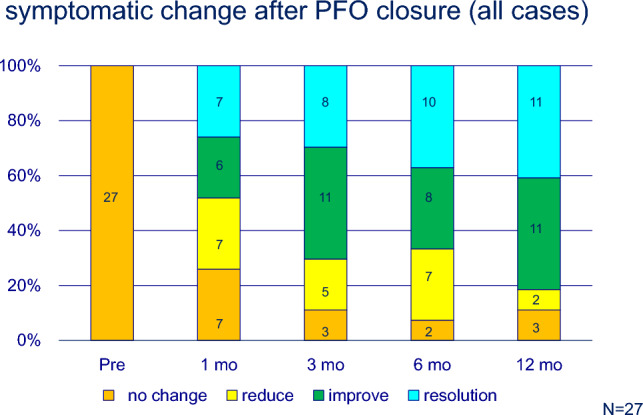
Symptomatic change after PFO closure in patients’ migraine

**Fig. 3:**
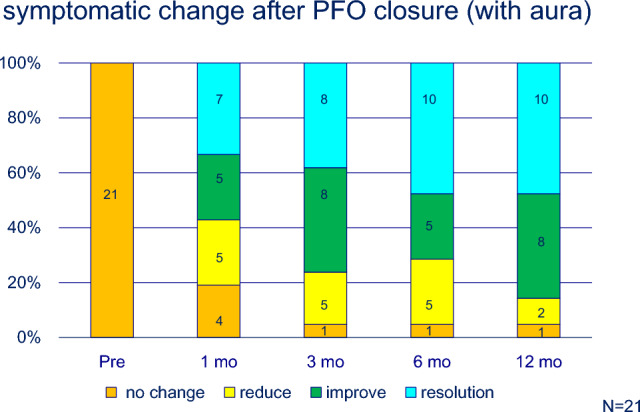
Symptomatic change after PFO closure in patients’ migraine with aura

**Fig. 4:**
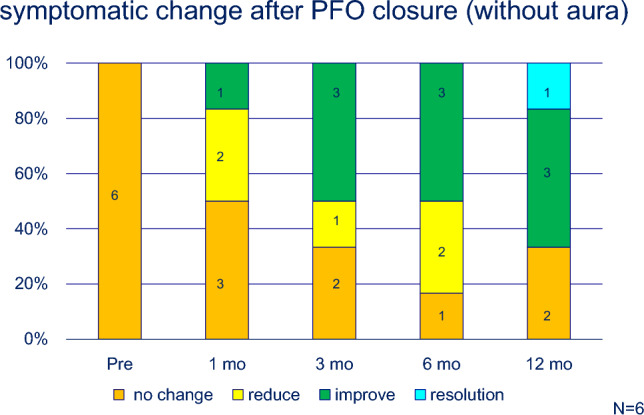
Symptomatic change after PFO closure in patients’ migraine without aura

No complications, including atrial arrhythmias or cardiac erosion, were observed. Migraine severity improved in most cases, with some cases showing complete resolution (Table 1). Approximately, 80% (22 of 27) of patients showed significant improvement or resolution after 1 year (Fig. 2). Patients with migraine with aura showed a more pronounced response; 10 of 21 (48%) patients experienced complete resolution after 1 year (Fig. 3). In contrast, the effectiveness of transcatheter closure was less clear in patients without aura, and only one patient experienced headache resolution 1 year after the procedure (Fig. 4). In this study, the limited sample size prevented evaluation of the relationship between PFO morphology and treatment efficacy.

## Discussion

Migraine remains a prevalent and challenging disorder despite advances in understanding and treatment. Approximately, 50% of patients with migraine with aura have PFO, suggesting an association, whereas no difference is noted in patients with migraine without aura compared to the general population [10, 22]. It is hypothesized that serotonin-related substances and microthrombi passing through PFO may trigger cortical spreading depression (CSD), leading to migraines [1423].

Although current migraine medical treatments provide symptom relief, they do not cure migraine and require ongoing medication [24]. Transcatheter PFO closure offers a distinct advantage as a single procedure with the potential to eliminate migraines [8]. However, previous trials have failed to demonstrate efficacy due to factors such as inclusion of patients resistant to medical therapy, insufficient sample sizes, and inadequate morphological assessments [23–25]. In this study, the efficacy of PFO closure was more evident in patients with migraine with aura. Nonetheless, some patients with migraine without aura also benefited, highlighting the heterogeneous nature of migraine.

This article is not intended to prove the efficacy of transcatheter PFO closure over conventional medical treatment. Medical treatment should always be the first choice for migraine treatment, and recent improvements in CGRP inhibitors have greatly improved treatment outcomes [14]. However, in the real world, long-term medical treatment requires patient tolerance, and a large number of patients take off-the-shelf medications repeatedly [12]. If transcatheter PFO closure can eliminate migraine with a single procedure, it will have major clinical implications [25].

Recently, transcatheter PFO closure has been widely used for the prevention of recurrent cryptogenic stroke, and considerable interest has been focused on the morphologic evaluation of PFO, which has been understudied [26]. PFO with atrial septal aneurysm, long tunnel, and large shunt are now recognized as high-risk PFO, as they have been shown to have a high risk of causing PFO-related stroke [18, 26]. This study could not conclude whether or not high-risk PFOs are more common in patients with migraine with aura. However, if it is possible to perform transcatheter PFO closure for the treatment of migraine in such high-risk PFO cases, this may lead to new clinical insights. In addition, this study may also lead to the discovery of the importance of transcatheter PFO closure as a primary prevention strategy for cryptogenic stroke.

### Limitations

This study was non-randomized small number single institutional study and contains various biases. The willingness of patients to undergo the procedure and bear the cost may have influenced their psychological state and perceived treatment outcomes. Although an independent neurologist evaluated migraine symptoms, bias cannot be completely excluded.

Morphological assessment of PFO was limited as the concept of high-risk PFO had not yet been established. In addition, no bubble studies were performed during follow-up to assess residual shunts. The temporary use of clopidogrel, which may reduce migraines [27], could have influenced early assessments but was unlikely to affect long-term outcomes.

## Conclusion

Despite these limitations, some patients experienced significant clinical effects, including complete resolution of migraines—a result difficult to achieve with drug therapy. Although current evidence for PFO closure in migraine treatment is insufficient, accumulating further data may help identify patients likely to benefit from this intervention.
